# Supplementary material for: Population variation in miRNAs and isomiRs and their impact on human immunity to infection
Source: Genome Biol. 2020 Jul 30;21:187. doi: 10.1186/s13059-020-02098-w (PMC7391576; doi:10.1186/s13059-020-02098-w)
Supplement: Supplementary file 1 — Additional file 1: Fig. S1. Quality and pre-processing of small RNA sequencing data. Fig. S2. IsomiR diversity and rate of miRNA modifications. Fig. S3. Sources of isomiR variation upon immune activation. Fig. S4. Genetic basis of miRNA expression. Fig. S5. Detection of miRNA-mRNA correlations. [file 13059_2020_2098_MOESM1_ESM.docx]

**Additional File 1**

Supplemental Figures S1-S5

**Population variation of miRNAs and isomiRs and their impact on human immunity to infection**

Maxime Rotival, Katherine J Siddle, Martin Silvert, Julien Pothlichet, Hélène Quach, Lluis Quintana-Murci

**
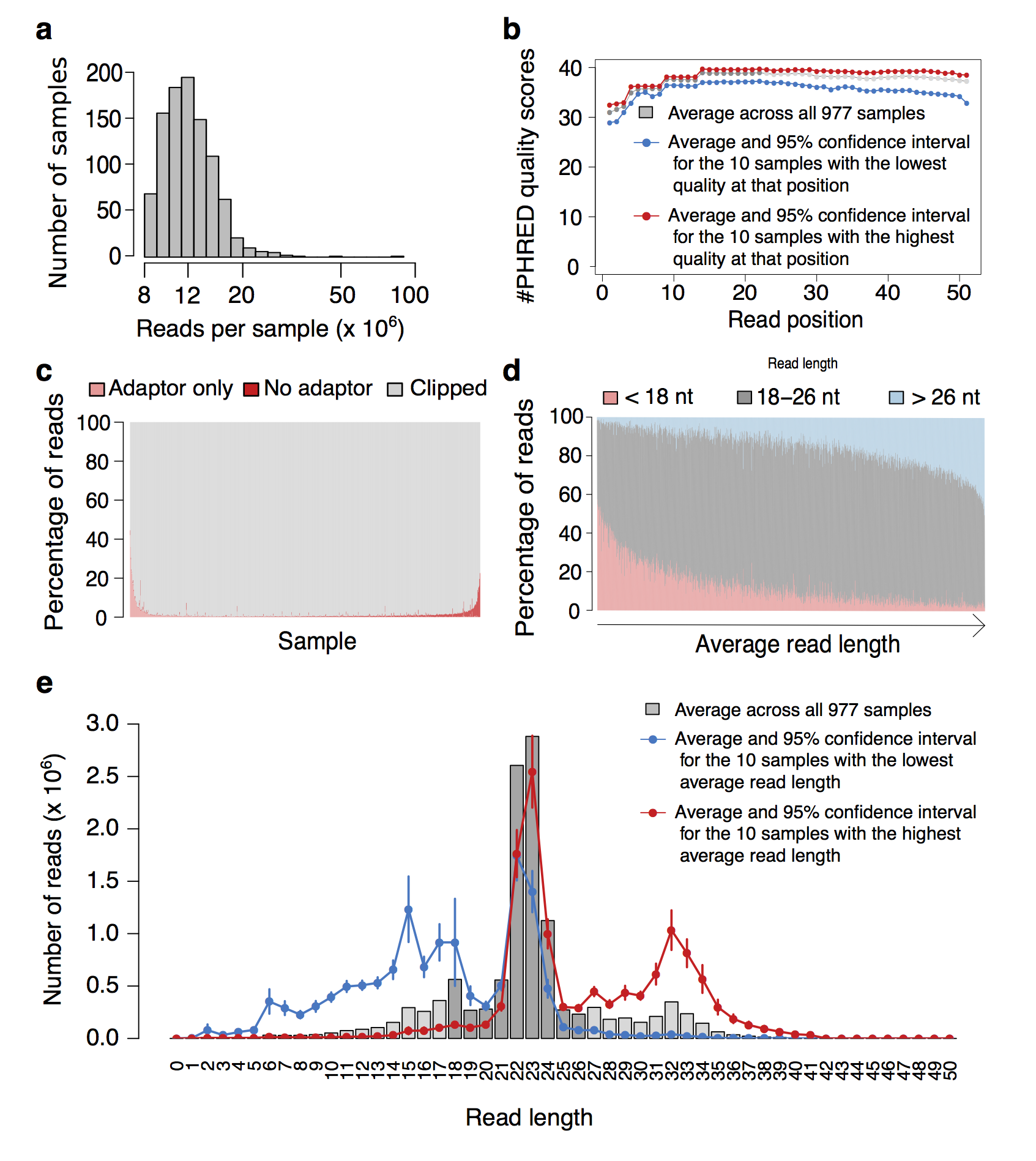
**

**Fig. S1. Quality and pre-processing of small RNA sequencing data**. **a** Histogram of the total number of sequenced reads per sample. **b** Mean quality of sequenced reads. For each position along the 50bp of the sequenced reads, the mean quality at that position (across all 977 samples) is reported in grey. In addition, at each position, the mean quality among the 10 samples with highest and lowest quality at that position is show in blue and red, respectively. Vertical bars indicate 2 standard errors from the mean among these samples. **c** Distribution, among the 977 high quality samples, of the fraction of reads removed during the adaptor trimming. Each column is one sample. Reads where only the adaptor was sequenced are displayed in red, and reads that lack of adaptor sequence and displayed in pink. Correctly clipped reads, are displayed in grey. **d** Distribution, among the 977 high-quality samples, of the fraction of reads removed based on length. Each column is one sample. Reads that are shorter than 18 nucleotides are displayed in red. Reads that are longer than 26 nucleotides are displayed in blue. Reads that are kept for downstream analyses are shown in grey. Samples are sorted according to their average read length. **e** Distribution of read lengths after adaptor trimming. For each possible read length, the mean number of read per sample is displayed as a grey bar (darker grey is used for the 18-26 nucleotide range). In addition, at each possible length, the mean number of reads among the 10 samples with highest and lowest average read length is shown in blue and red, respectively. Vertical bars indicate 2 standard errors from the mean, among these samples.

**
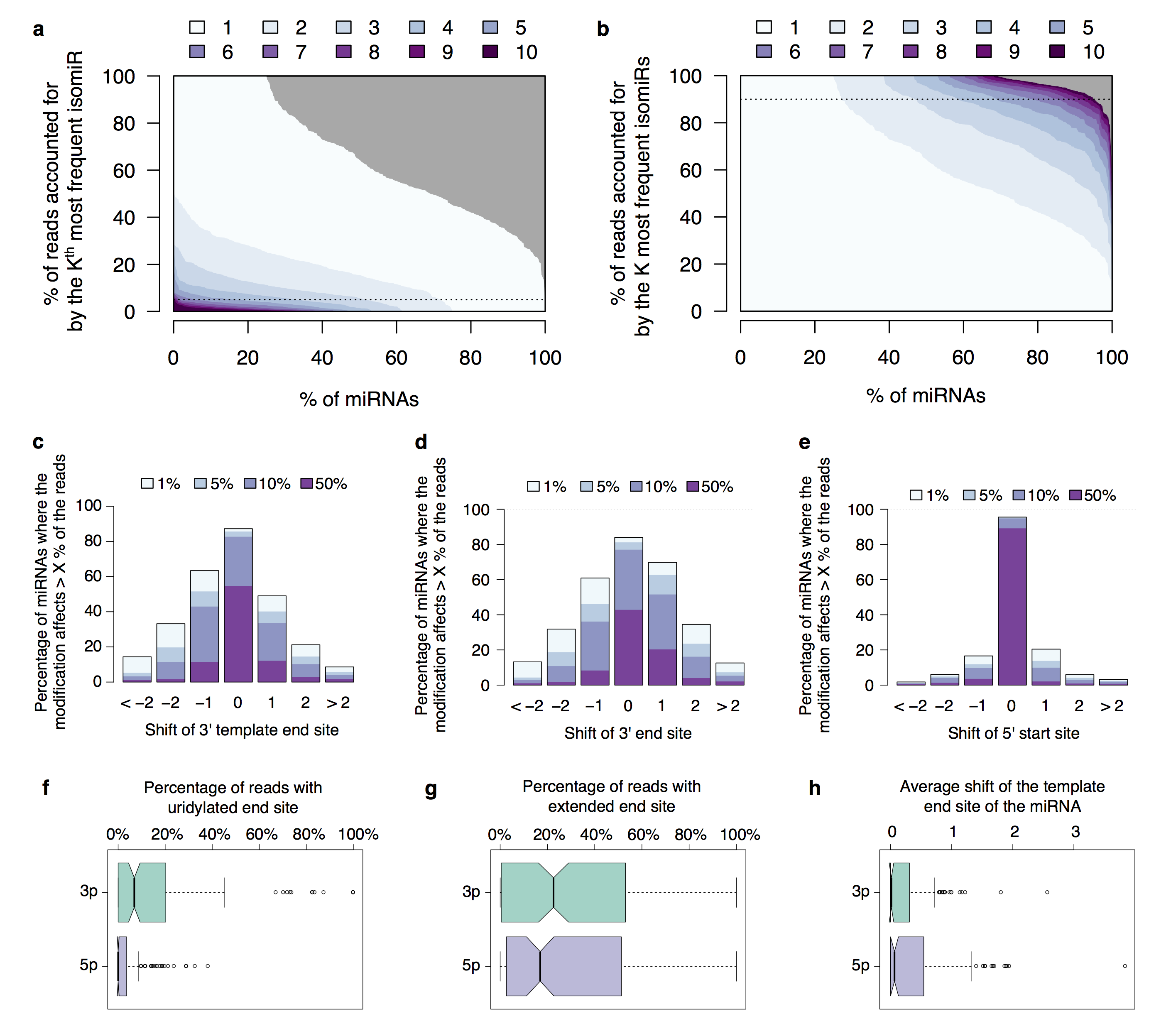
**

**Fig. S2.** **IsomiR diversity and rate of miRNA modifications**. **a** Distribution of the percentage of reads accounted by the 10 most frequent isomiRs across miRNAs. **b** Distribution of the cumulative percentage of reads accounted by the 10 most frequent isomiRs. **c** Distribution of shifts in 3’ end site, excluding non-template additions. For each possible shift of the 3’ end site, we report the percentage of miRNAs for which the corresponding isomiRs accounts for at least 1% (light blue), 5% (blue), 10% (indigo) and 50% (deep purple) of edited reads. **d** Distribution of shifts in 3’ end site, including non-template additions. **e** Distribution of shifts in 5’ start site. **f** Average percentage of miRNA reads uridylated at their 3’ end, for 3p miRNAs (green) and 5p miRNAs (purple). **g** Average percentage of miRNA reads with an extended 3’ end site, for 3p miRNAs (green) and 5p miRNAs (purple). **h** Average shift of the 3’ end site, for 3p miRNAs (green) and 5p miRNAs (purple), excluding non-template additions.

**
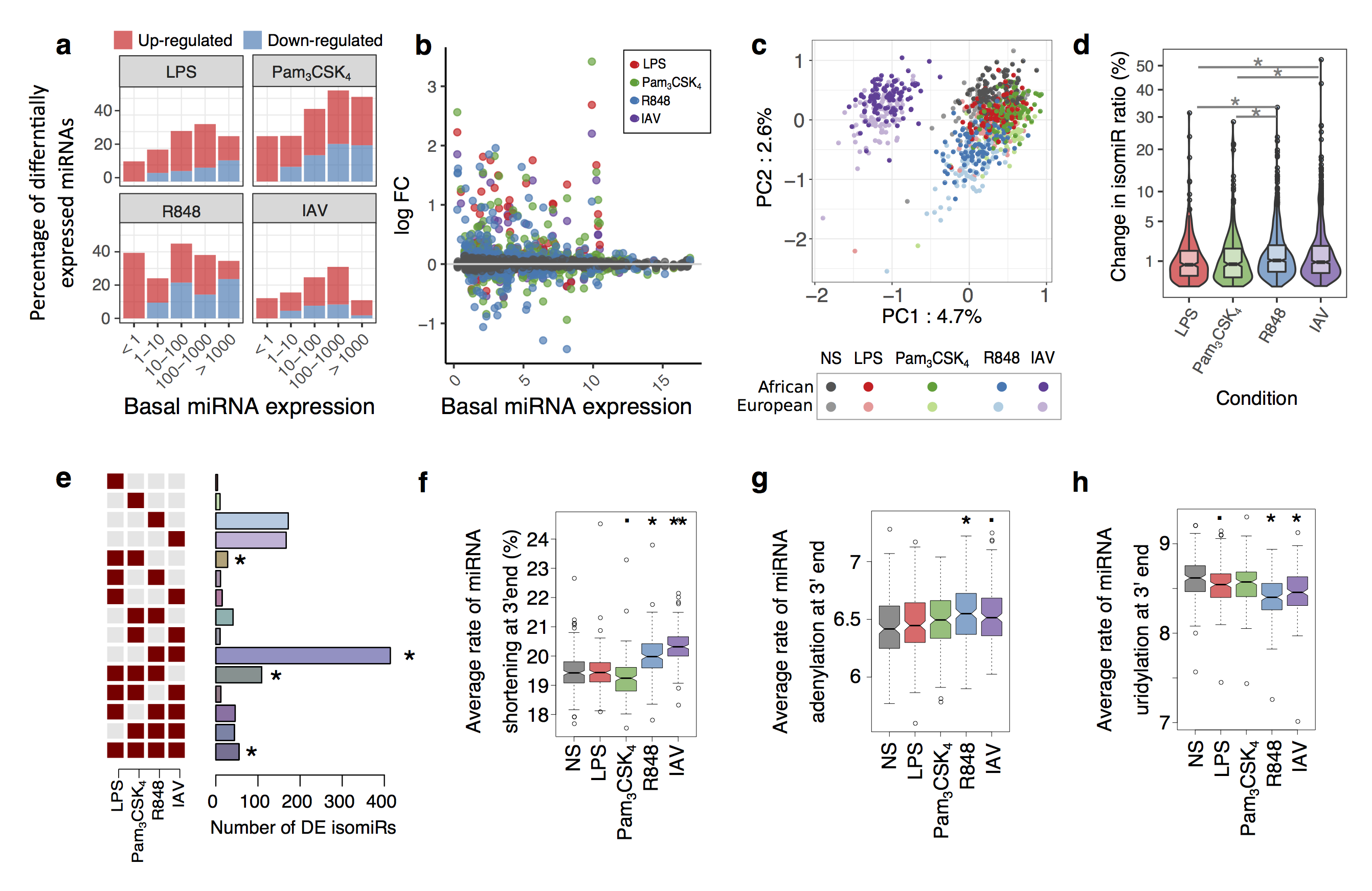
**

**Fig. S3. Sources of isomiR variation upon immune activation**. **a** Percentage of differentially expressed (DE) miRNAs between non-stimulated and stimulated conditions, according to the basal level of miRNA expression. For each bin and condition, DE miRNAs are split between up- and down-regulated (red and blue, respectively). **b** Fold change in gene expression upon stimulation as a function of basal miRNA expression. Each stimulation condition is represented by a distinct color (red – LPS, green – Pam_3_CSK_4_, blue – R848, purple – IAV; the same color code for conditions is used throughout the manuscript). miRNAs that do not change significantly their expression are shown in dark grey. **c** PCA of the ratios of commons isomiR (RPM>1, >1% of miRNA reads). Each dot represents a sample, colored according to the condition of stimulation (grey – non-stimulated, stimulated conditions – see above), light and dark shades indicate African and European ancestry, respectively. **d** For each condition, violin plots showing the distribution of absolutes changes in isomiR ratios for DE isomiRs. (*Wilcoxon *p*<.01) **e** Number of isomiRs that change their ratio (compared to NS) in a single stimulus or a combination of stimuli (*binomial *p* < 0.001, significance of overlap between stimuli). **f** For each sample, the rate of miRNA shortening at the 3’ end was obtained as the average across all miRNAs of the percentage of isomiRs that have a shortened 3' end (symbols indicate significant deviations from the non-stimulated state based on Wilcoxon rank test; •*p*_adj_<0.05, **p*_adj_<0.01, ***p*_adj_<10^-20^). **g-h** For each sample, the rate of miRNA adenylation (**g**) and uridylation (**h**) at the 3’ end was obtained as the average across all miRNAs of the percentage of isomiRs that are adenylated or uridylated at their terminal site (symbols indicate significant deviations from the non-stimulated state based on Wilcoxon rank test; (•*p*_adj_<0.05, * *p*_adj_<0.01).


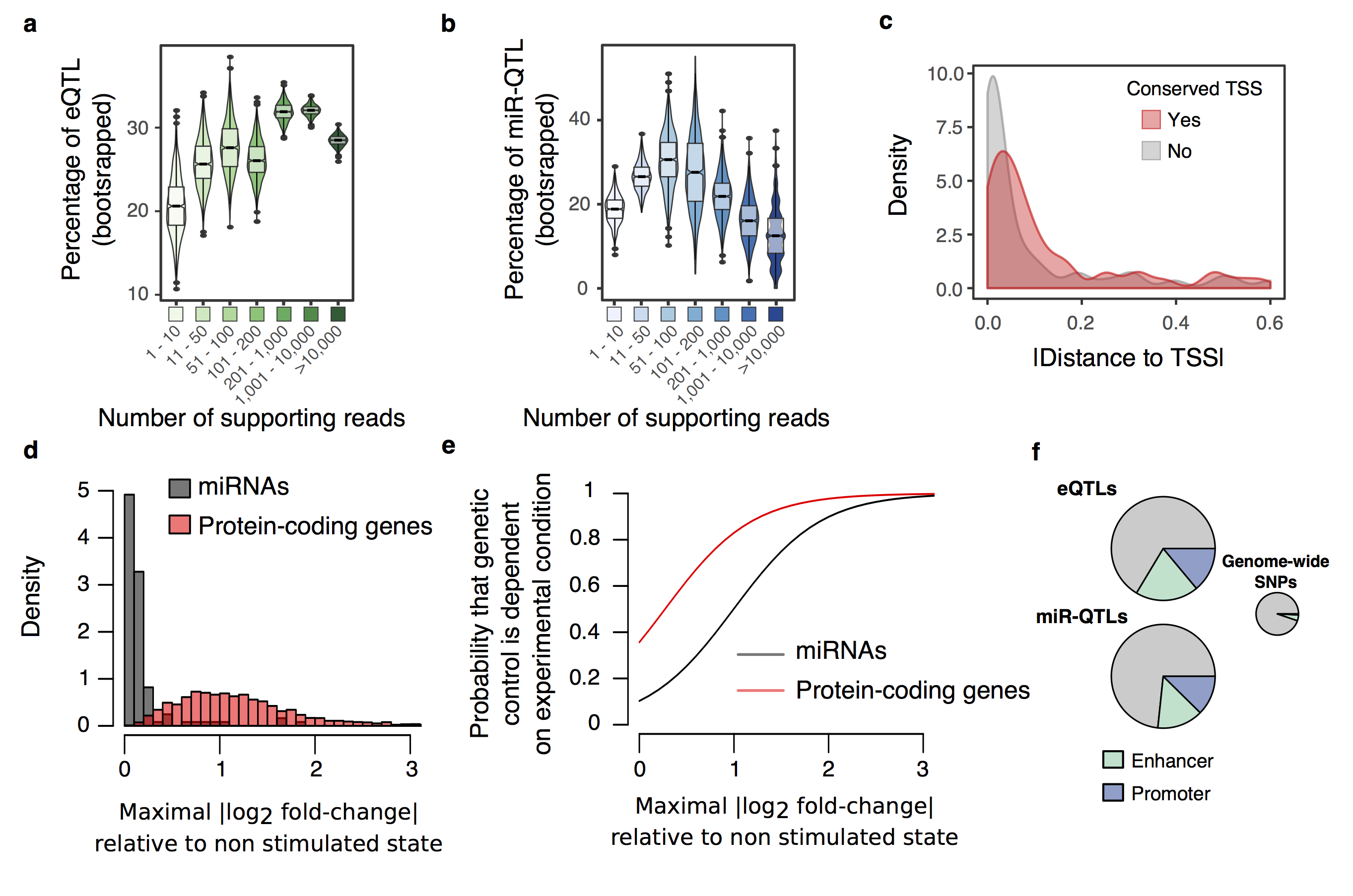


**Fig. S4. Genetic basis of miRNA expression**. **a** Percentage of protein-coding genes with an eQTL, for various levels of read coverage (measured in number of supporting reads per sample). For each gene group, 1000 bootstrap resamples were performed and the resulting distribution is shown as a boxplot. **b** Same as **a** for miRNAs. **c** Distance of miR-QTLs from their associated transcription start site (TSS). Density plots showing the distribution of distance in Mb between miR-QTLs and the transcription start site of their associated pri-miRNA. Distribution is shown separately for miRNAs with conserved (red, mean phastCons>20%) and non-conserved (grey, mean phastCons<20%) promoters. **d** Distribution of maximal absolute log_2_ fold changes in expression after stimulation, for miRNAs and protein-coding genes. **e** Estimated probability of condition-dependent genetic control for miRNAs and protein-coding genes, as a function of maximal absolute log_2_ fold change in expression upon stimulation. **f** Percentage of eQTLs and miRQTLs overlapping annotated enhancers or promoters. Percentage of frequent SNPs (MAF>5%) overlapping enhancers and promoters, at the genome-wide scale, is provided as a reference.

**
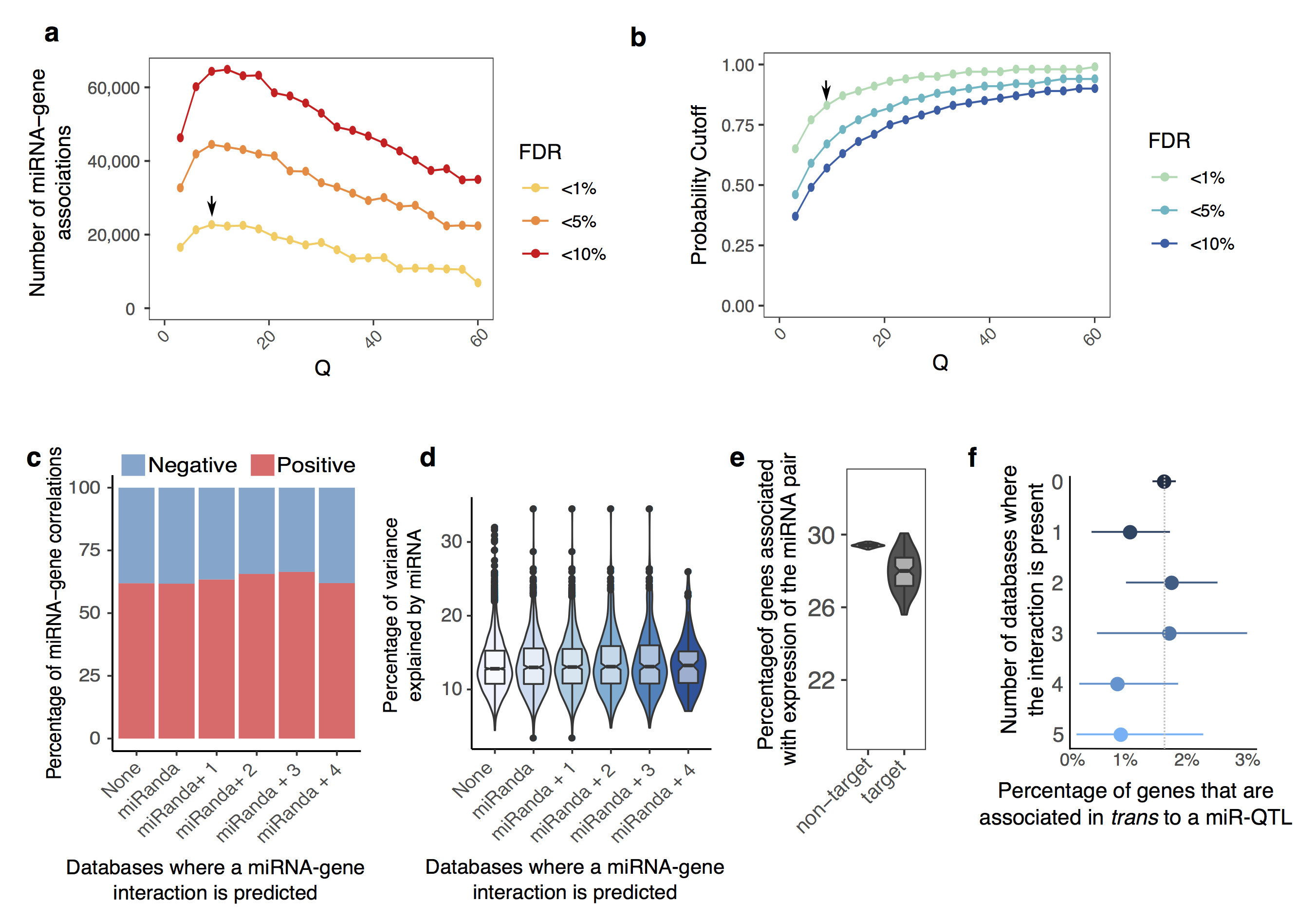
**

**Fig. S5. Detection of miRNA-mRNA correlations**. **a** Number of detected miRNA-gene associations according to the Q parameter, controlling the maximal number of miRNAs used as predictors of gene expression, and the required level of FDR. **b** Probability cut-off yielding a 1%, 5% and 10% FDR according to the value of the Q parameter. **c** Percentage of miRNA-gene pairs that are either positively or negatively correlated according to the presence or absence of a miRanda-predicted miRNA binding site, and to the number of databases that support the binding. **d** Distribution of the percentage gene variance explained by associated miRNAs according to the presence or absence of a miRanda-predicted miRNA binding site, and to the number of databases that support the binding. **e** Estimated percentage of genes that are associated with a pair of interacting miRNAs, among genes with a pair of co-localized targets (*target*) and in the rest of the genome (*non-target*). For each group, the distribution shows the uncertainty based on 100 bootstrap replicates. **f** Estimated percentage of genes associated in *trans* to a miR-QTL or isomiR-QTL, according to the presence/absence of a binding site for the associated miRNA, and the number of databases where this binding site is predicted.
